# Deconvoluting drug interactions using *M. tuberculosis* physiologic processes: transcriptional disaggregation of the BPaL regimen *in vivo*

**DOI:** 10.1128/aac.00492-25

**Published:** 2025-09-18

**Authors:** Elizabeth A. Wynn, Christian Dide-Agossou, Reem Al Mubarak, Karen Rossmassler, Jo Hendrix, Martin I. Voskuil, Andrés Obregón-Henao, Michael A. Lyons, Gregory T. Robertson, Camille M. Moore, Nicholas D. Walter

**Affiliations:** 1 Center for Genes, Environment and Health, National Jewish Health 2930, Denver, Colorado, USA; 2 Rocky Mountain Regional VA Medical Center 19982 , Aurora, Colorado, USA; 3 Consortium for Applied Microbial Metrics, Aurora, Colorado, USA; 4 Division of Pulmonary Sciences and Critical Care Medicine, University of Colorado Anschutz Medical Campus 129263, Aurora, Colorado, USA; 5 Linda Crnic Institute for Down Syndrome, University of Colorado Anschutz Medical Campus 637889, Aurora, Colorado, USA; 6 Department of Immunology and Microbiology, University of Colorado Anschutz Medical Campus 129263, Aurora, Colorado, USA; 7 Mycobacteria Research Labs, Department of Microbiology, Immunology, and Pathology, Colorado State University 3447, Fort Collins, Colorado, USA; 8 Department of Biostatistics and Informatics, University of Colorado Anschutz Medical Campus 129263, Aurora, Colorado, USA; City St George's, University of London, London, United Kingdom

**Keywords:** RNA sequencing, antibiotic interaction, *M. tuberculosis*, antibiotic exposure

## Abstract

A key challenge in preclinical tuberculosis drug development is identifying optimal antibiotic combinations. Drug interactions are complex because one drug may affect *Mycobacterium tuberculosis* (*Mtb*) physiology in a way that alters the activity of another drug. Conventional pharmacodynamic evaluation based on colony-forming units (CFU) does not provide information about this physiologic interaction because CFU enumerates bacteria but does not give information about the drug’s effect on bacterial cellular processes. SEARCH-TB is a novel pharmacodynamic (PD) approach that uses targeted *in vivo* transcriptional profiling to evaluate drug effects on *Mtb* physiology. To evaluate SEARCH-TB’s capacity to elucidate drug interactions, we deconstructed the BPaL (bedaquiline, pretomanid, and linezolid) regimen in the BALB/c high-dose aerosol mouse infection model, measuring the effect of 2-, 7-, and 14-day treatment with drugs in monotherapy, pairwise combinations, and as a three-drug combination. Monotherapy induced drug-specific *Mtb* transcriptional responses by day 2 with continued evolution over 14 days. Bedaquiline dominated pairwise combinations with pretomanid and linezolid, whereas the pretomanid-linezolid combination induced a transcriptional profile intermediate between either drug. In the three-drug BPaL regimen, adding both pretomanid and linezolid to bedaquiline yielded a greater transcriptional response than expected, based on pairwise results. This work demonstrates that physiologic perturbations induced by a single drug may be modified in complex ways when drugs are combined. This establishes proof of concept that SEARCH-TB provides a highly granular readout of drug interactions *in vivo,* providing information distinct from CFU burden and suggesting a future where regimen selection is informed by *in vivo* molecular measures of *Mtb* physiology.

## INTRODUCTION

Tuberculosis (TB) is an ongoing public health crisis, causing ~10 million illnesses and ~1.4 million deaths each year (1). TB requires prolonged multi-drug treatment (2). To control TB, there is an urgent need for new regimens that cure both drug-susceptible and drug-resistant TB more quickly. Mouse treatment models play a crucial role in the preclinical evaluation of drugs and combination regimens (3–5). In murine TB drug evaluation, drug effects are measured based on pharmacodynamic (PD) markers. The US Food and Drug Administration and European Medicines Agency definition of a PD biomarker is “a response marker that indicates biological activity of a medical product (6, 7).” Here, we describe an early step in establishing a novel type of PD marker for TB drug evaluation.

In recent years, the number of promising individual new drugs and candidate compounds in the TB drug development pipeline has expanded (8–10). A key challenge is identifying combinations of these drugs that maximize efficacy. Unfortunately, conventional PD tools such as colony-forming units (CFU) cannot reliably predict the efficacy of a drug combination based on the activity of individual drugs. Depending on their mechanism of action, drugs injure *Mycobacterium tuberculosis* (*Mtb*) differently, causing drug mechanism-specific physiologic perturbations (11–13). Since drug activity depends on the physiologic state of the bacterium (14–16), the effect of one drug may change the physiologic state of *Mtb* in a way that either favors the activity of a second antibiotic (synergy) or disfavors the activity of a second antibiotic (antagonism).

A limitation compounding the understanding and prediction of combinatorial effects is that drug interactions are generally classified based on crude, culture-based measures of bacterial burden. Minimum inhibitory concentration (MIC) and minimum bactericidal concentration (MBC) (13) estimate the threshold concentration at which bacterial burden remains constant or decreases by 99%, respectively. Similarly, CFU, the PD marker conventionally used in animal studies, enumerates *Mtb* burden. PD markers of bacterial burden (e.g*.,* CFU) indicate only the presence or absence of the *Mtb,* providing no information about the complex processes of injury and adaptation that are initiated by drug binding with its molecular target or that occur when drugs interact. More than 70 years ago, Eagle hypothesized that bacteria that are not immediately killed by drugs sustain damage to essential physiologic processes (17), initiating a “cascade of injury” that augments over time as homeostatic mechanisms are progressively disrupted. *Mtb* is also capable of adapting physiologic processes to withstand stresses, including drug exposure. Because these effects are not measured by CFU, there is, therefore, a need for adjunctive PD tools that can complement CFU by providing information about drug effects on bacterial physiology in preclinical animal studies.

The effect of drugs on bacterial physiologic state can be measured by transcriptional profiling. The effect of antibiotics on *Mtb* transcription has been studied extensively in short-term *in vitro* experiments (18–24), but short-term exposure in axenic culture may not replicate the physicochemical conditions and dynamic pharmacokinetics encountered during chronic *in vivo* exposure. For the purpose of characterizing drug effects in animal models*,* we recently developed SEARCH-TB, a targeted RNA-sequencing (RNA-seq) platform (11, 25). SEARCH-TB uses a novel combination of selective eukaryotic cell lysis to deplete host RNA, followed by targeted *Mtb* mRNA amplification. Comparison with prior approaches (Supplemental Section 2.5 of our methods manuscript [25]) showed that SEARCH-TB has breakthrough sensitivity enabling high-granular quantification of low-abundance *Mtb* transcripts in lung tissue of mice treated with potent drug combinations. We have initiated a stepwise development process with the end goal of establishing SEARCH-TB as a practical and informative PD marker for preclinical drug and regimen evaluation in murine studies. We began by describing how standard isoniazid, rifampin, pyrazinamide, ethambutol (HRZE) four-drug regimen (25) and drugs in monotherapy (11) affect *Mtb* physiology *in vivo*. Here, we extend the descriptive phase of our work by evaluating the capacity of SEARCH-TB to elucidate drug interactions in the BALB/c high dose aerosol subacute TB mouse model.

Historically, TB regimens have been selected based on a “mixing and matching” process in which the effect of iterative individual drug substitutions on burden or relapse outcomes is observed and compared. This empirical process led to the existing global standard four-drug regimen for drug-susceptible TB (HRZE) (26) and is used in contemporary human trials and murine studies. Unfortunately, this approach is time-consuming and can evaluate only a small fraction of possible drug/dose combinations (27). To move from empirical mix and match testing to rational regimen design, there is an unmet need for a PD marker that provides information about the effect of drug interactions on *Mtb* physiologic state *in vivo*. Our end goal is to develop SEARCH-TB as a PD tool for highly granular assessment of drug and regimen effects *in vivo*. As an initial step toward that longer-term goal, here, we tested the capacity of SEARCH-TB to probe drug interactions *in vivo* and compared this information with CFU.

We used SEARCH-TB to evaluate interactions among drugs in the bedaquiline, pretomanid, linezolid (BPaL) regimen, which recently revolutionized treatment of drug-resistant TB (28, 29). Conducting a time-course study in the BALB/c mouse treatment model, which is a preclinical reference standard (30), we compared the effect of drugs individually, pairwise, and as a three-drug combination on the physiologic state of *Mtb*. The novel SEARCH-TB *in vivo* physiologic readout revealed that drugs with different mechanisms of action resulted in a distinct pattern of injury and adaptation that evolved with increasing treatment duration. In pairwise interactions, the effect of bedaquiline dominated interactions. In the full three-drug regimen, the combination of pretomanid and linezolid made a transcriptionally synergistic contribution to bedaquiline.

## RESULTS

### Effect of individual drugs and combinations on CFU

**Fig 1:**
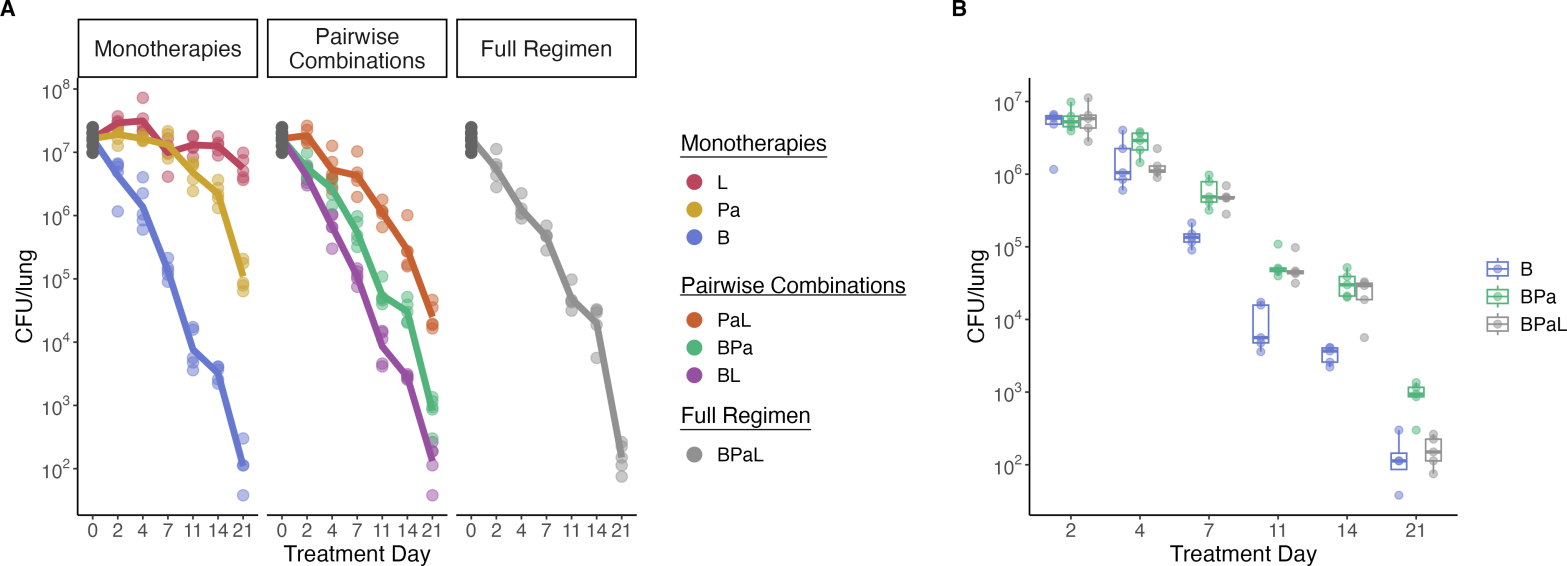
Effect of individual drugs and combinations on CFU. (**A**) CFU burden over time in the lungs of BALB/c mice after treatment with bedaquiline (**B**), pretomanid (Pa), linezolid (L), and all combinations of the antibiotics. Points indicate CFU values from individual mice. For each treatment condition, the lines connect the average value at each time point. (**B**) Direct comparison of CFU for bedaquiline (B), BPa, and BPaL across time.

In the standard BALB/c mouse high-dose aerosol infection model, we first quantified the conventional PD marker (lung CFU counts) during treatment with individual drugs, pairwise drug combinations, and the three-drug combination of bedaquiline, pretomanid, and linezolid. Given as monotherapy, bedaquiline decreased the median CFU by 99.2% (2.1 log_10_ difference) from pretreatment control by day 7 and 99.999% (5.17 log_10_ difference) by day 21 (Fig. 1A). Pretomanid reduced the CFUs more gradually by 27.78% (0.14 log_10_ difference) by day 7 and 99.49% (2.29 log_10_ difference) by day 21. Linezolid had a diminutive effect, reducing CFU by 33.33% (0.18 log_10_ difference) by day 7 and by 70% (0.52 log_10_ difference) by day 21. In pairwise combinations, linezolid did not discernibly add to the bactericidal effect of bedaquiline. The addition of linezolid to pretomanid did significantly increase bactericidal activity relative to pretomanid alone (*P*-values < 0.02 at days 7, 11, 14, and 21). As previously observed (31, 32), the combination of pretomanid and bedaquiline had significantly less bactericidal activity than bedaquiline alone (Fig. 1B, *P*-values <0.01 at days 7, 11, 14, and 21). Similarly, the BPaL regimen had significantly less effect on CFU than bedaquiline alone on days 7, 11, and 14 (*P*-values < 0.003), although the three-drug regimen and monotherapy were indistinguishable at day 21 (*P*-value = 0.549) (Fig. 1B).

### Cascade of injury and adaptation initiated by individual drug exposures

**Fig 2:**
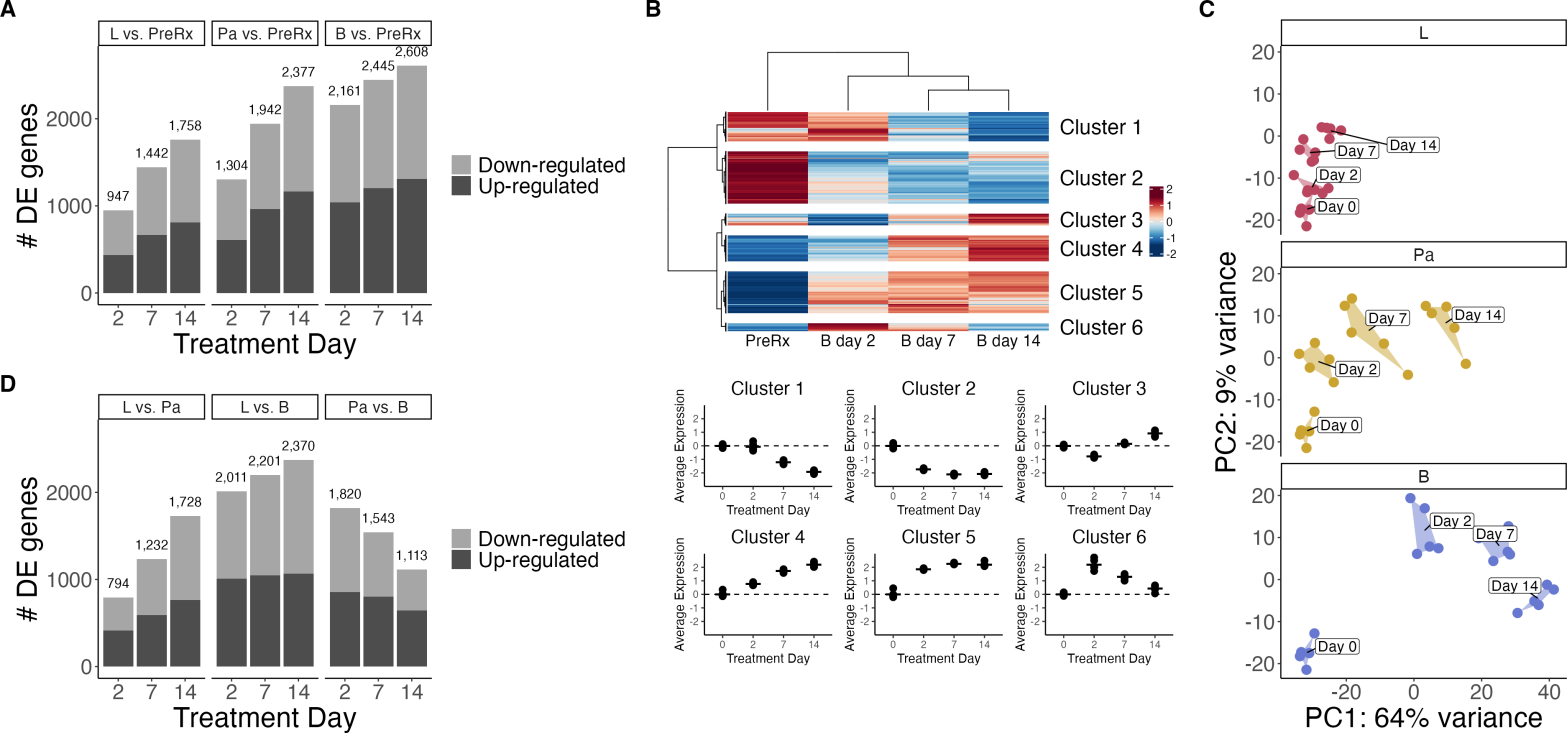
The effect of monotherapies on gene expression. (**A**) Number of differentially expressed genes for bedaquiline (B), pretomanid (Pa), or linezolid (L) compared with pretreatment control at each time point. (**B**) Hierarchical clusters of genes differentially expressed over time with bedaquiline treatment. The heatmap shows the batch-adjusted, VST-normalized, scaled gene expression averaged across samples. Clustering identified six broad patterns. Dot plots show the average expression for each cluster across time points. Each point represents an individual mouse. Horizontal lines indicate average values. Values are centered around the average value for the pretreated samples so that points above and below zero represent upregulation and downregulation relative to pretreatment, respectively. (**C**) The first two principal components of batch-adjusted VST-normalized gene expression data for the top 500 most variable genes. PCs were calculated across all experimental conditions. Shown are samples from monotherapy-treated mice across time. Each point represents an individual sample, and a convex hull highlights each time point. (**D**) Number of differentially expressed genes between each pair of the individual antibiotics at each time point.

Next, we evaluated the effect of individual drugs on the *Mtb* transcriptome in the same experiment described above, using SEARCH-TB at treatment days 0, 2, 7, and 14. Monotherapy with bedaquiline, pretomanid, or linezolid induced a rapid and profound transcriptional response, significantly altering the expression of 2,161 (60.6%), 1,304 (36.5%), and 947 (26.5%) *Mtb* genes, respectively, relative to the pretreatment control after 2 days of dosing (Fig. 2A). Genes with a Benjamini–Hochberg adjusted *P*-value (33) less than 0.05 were considered significant. With continued treatment, the *Mtb* transcriptional response to drug injury evolved further. For example, hierarchical clustering of differentially expressed genes revealed that bedaquiline exposure caused distinct clusters of genes to change concordantly over time (Fig. 2B). We performed gene set enrichment on each cluster using gene categories from Cole et al. (34) and curated from the literature (Table S2), with enrichment assessed using hypergeometric testing with Benjamini-Hochberg adjusted *P*-values less than 0.05 denoting significance. Cluster 1 consisted of 500 genes that had initially stable expression at day 2, followed by decreased expression at days 7 and 14, and was significantly enriched for ESX1 and ESX3, which are involved in active manipulation of host response (adj-p <0.001, File S1). Cluster 2 consisted of 887 genes with progressively decreased expression and was enriched for cell wall synthesis (adj-p <0.001), as well as categories involved in translation (e.g., primary ribosomal proteins, adj-p <0.001) and metabolism (e.g., aerobic respiration, adj-p <0.001), indicating that bedaquiline-induced ATP starvation caused a global slowdown in cellular activity. Clusters 4 (443 genes) and 5 (708 genes) had progressively increased expression over time. In addition to generalized stress responses including insertion sequences and phage-related function (adj-p = 0.015), Cluster 5 was enriched for genes involved in regulatory function and specific responses that we have previously observed during adaptation to drug stress (11), including ESX4 (adj-p = 0.011), Mce3 (adj-p <0.001), and fumarate reductase genes (adj-p = 0.024). Cluster 3 contained 199 genes that had decreased expression on day 2 and increased thereafter, whereas cluster 6 contained 138 genes that increased on day 2 and decreased thereafter. Cluster 6 was enriched for transcription factors (adj-p = 0.002) and other regulatory elements, consistent with a transient reprogramming with the onset of drug stress. Linezolid and pretomanid also caused progressive transcriptional change as illustrated in Fig. S1; Files S2 and S3.

As we previously observed with first-line TB drugs (11), the patterns of injury and adaptation induced by bedaquiline, pretomanid, and linezolid were distinct both in terms of the magnitude and pattern of expression change. To visualize these differences, we performed principal component analysis (PCA) across all monotherapies and regimens at all time points and plotted the results on a single PCA plot (Fig. S2). Subsetting this plot to display each drug individually revealed that each drug led to a different *Mtb* transcriptional profile over time (Fig. 2C). Direct comparison between drug-stressed phenotypes identified large numbers of differentially expressed genes (Fig. 2D). Over time, the effects of bedaquiline and pretomanid grew progressively more distinct from linezolid. The number of differentially expressed genes relative to linezolid increased from 2,011 to 2,370 for bedaquiline and from 794 to 1,728 for pretomanid between days 2 and 14, respectively. Conversely, over time, the effects of bedaquiline and pretomanid grew more similar, with the number of differentially expressed genes decreasing from 1,820 to 1,113 from day 2 to day 14.

**Fig 3:**
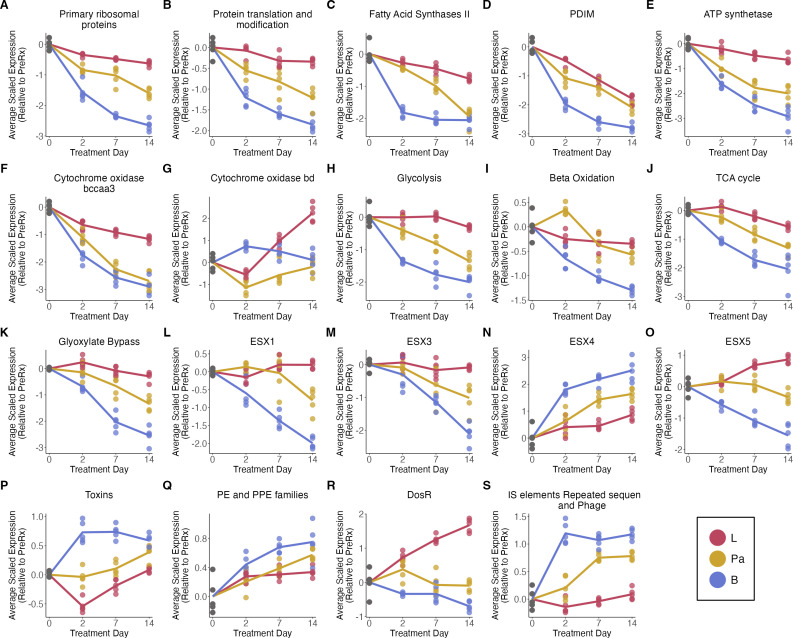
Effect of monotherapies on key Mtb biological processes. Average of batch adjusted, VST-normalized, scaled gene expression in each monotherapy over time for genes involved in: (**A**) primary ribosomal proteins, (**B**) protein translation and modification, (**C**) fatty acid synthases II, (**D**) PDIM, (**E**) ATP synthetase, (**F**) cytochrome oxidase bccaa3, (**G**) cytochrome oxidase bd, (**H**) glycolysis, (**I**) beta-oxidation, (**J**) TCA cycle, (**K**) glyoxylate bypass, (**L**) ESX-1, (**M**) ESX-3, (**N**) ESX-4, (**O**) ESX-5, (**P**) toxins, (**Q**) PE and PPE families, (**R**) dosR, and (**S**) IS elements, repeated sequences, and phage. Each point represents an individual mouse, and the lines connect the mean for each time point. Values are centered around the average value for the pretreated samples so that points above and below zero represent upregulation and downregulation relative to untreated, respectively.

We then interrogated the expression of the established gene categories from Cole et al. (34) and curated from the literature (Table S2). We summarized aggregate change for each category by calculating the average normalized expression of genes in the category for each mouse (Fig. S6; Fig. 3) and tested for differences between drugs and the pretreatment control and between the drugs themselves (exact *P-*values for all comparisons are included in File S4). Because the results presented here represent a subset of the transcriptional differences between drugs, the reader is encouraged to access full results via the Online Analysis Tool (https://microbialmetrics.org/analysis-tools/).

#### Suppressed expression of genes associated with protein translation

All antibiotics progressively suppressed the expression of primary ribosomal protein synthesis genes (Fig. 3A) and genes responsible for translational initiation, promotion of tRNA binding, elongation, termination, and protein folding (protein translation and modification category) (Fig. 3B). Suppression of both translation-related gene sets was significantly greater for bedaquiline on day 14 than for pretomanid (adj-p <0.01) or linezolid (adj-p <0.001) and significantly less for linezolid on day 14 than for pretomanid (adj-p <0.001).

#### Decreased synthesis of cell wall constituents

All antibiotics progressively suppressed the expression of fatty acid synthase II (FAS-II) genes central to cell wall mycolic acid synthesis (Fig. 3C). Decrease in FAS-II expression was more rapid for bedaquiline, with significantly lower expression on day 2 than for other drugs (adj-p <0.001). For pretomanid, FAS-II expression declined more slowly but eventually became statistically indistinguishable from bedaquiline by day 14. For linezolid, FAS-II declined to a lesser degree, sustaining significantly higher expression than bedaquiline or pretomanid at day 14 (adj-p < 0.001). All antibiotics progressively suppressed the expression of Phthiocerol dimycocerosate (PDIM), cell surface glycolipids that are virulence factors (Fig. 3D). The decrease in PDIM expression was significantly faster and more profound for bedaquiline than for other drugs (adj-p < 0.01 for all time points).

#### Metabolic slowing and adaptation

All antibiotics progressively suppressed the expression of genes coding for ATP synthetases (Fig. 3E), signaling decreased energy production. At day 14, bedaquiline suppressed ATP synthase gene expression significantly more than pretomanid (adj-p = 0.027) or linezolid (adj-p <0.001), and linezolid suppressed the expression significantly less than pretomanid (adj-p <0.001). All antibiotics progressively suppressed the expression of genes for the primary cytochrome *bcc/aa3* supercomplex, consistent with slowing of oxidative phosphorylation (Fig. 3F). Notably, this effect was significantly smaller for linezolid compared with other drugs (adj-p <0.001). Conversely, all drugs had sustained or increased expression of the less-efficient alternative cytochrome *bd* oxidase, which has been implicated in persistence under environmental and antibiotic stress (35). At day 14, this shift to cytochrome *bd* oxidase was significantly greater for linezolid than for other drugs (adj-p <0.001) (Fig. 3G). Slowing of respiration was further evidenced by the decreased expression of genes associated with glycolysis (Fig. 3H), beta-oxidation (Fig. 3I), and the TCA cycle (Fig. 3J). For all of these categories, bedaquiline suppressed the expression to a significantly greater degree at day 14 than pretomanid (adj-p <0.03) or linezolid (adj-p <0.001), consistent with metabolic quiescence. Bedaquiline (adj-p <0.001) and pretomanid (adj-p <0.001) suppressed the expression of glyoxylate bypass genes significantly on day 14 (Fig. 3K), signaling metabolic downregulation. By contrast, for linezolid, glyoxylate bypass gene expression did not change significantly relative to the control.

#### Expression of immunogenic secretory proteins

Antibiotics had different effects on the expression of ESX Type VII secretion systems that transport proteins across the mycobacterial cell wall. By day 14, the expression of genes in the ESX-1 locus, which exports the strongly immunogenic ESAT-6 and CFP-10 proteins, was suppressed by bedaquiline significantly more than other drugs (adj-p <0.001) (Fig. 3L). Linezolid did not significantly alter the ESX-1 expression relative to pre-treatment control. Similarly, expression of ESX-3, which also modulates host response and is essential for growth in activated macrophages, was suppressed by bedaquiline significantly more than pretomanid (adj-p = 0.029) and linezolid (adj-p <0.001) at day 14 (Fig. 3M). Linezolid did not significantly alter ESX-3 expression relative to pre-treatment control. Notably, at day 14, bedaquiline and pretomanid significantly induced the expression of ESX-4 genes (adj-p <0.001), a transporter of unknown function that was previously observed to be induced by HRZE (25) and other individual drugs (11) (Fig. 3N). Linezolid did not significantly alter ESX-4 expression relative to pre-treatment control. Expression of ESX-5 genes was affected differently by each drug, decreasing significantly for bedaquiline (day 14 adj-p <0.001), rising significantly for linezolid (day 14 adj-p <0.001), and remaining unchanged from control for pretomanid (Fig. 3O).

#### Regulation of growth: sigma factors

All antibiotics suppressed the expression of *sigA*, which codes for the primary “housekeeping” sigma factor necessary for growth. At every time point, suppression of *sigA* was significantly greater for bedaquiline than pretomanid (adj-p <0.04) or linezolid (adj-p <0.005), signaling a more quiescent phenotype. The 12 alternative sigma factors that regulate *Mtb* stress response were expressed in patterns specific to each drug. For example, in heatmaps showing unsupervised clustering of sigma factor gene expression, samples typically clustered by drug (Fig. S3A through C).

#### Modulation of stress responses

Antibiotics had distinct effects on the expression of genes for toxins that act post-transcriptionally to reprogram *Mtb* in response to stress (Fig. 3P). Bedaquiline induced toxin expression rapidly and significantly (adj-p <0.001 for all time points). Pretomanid induced toxin expression significantly more slowly, eventually rising to become statistically indistinguishable from bedaquiline at day 14. By contrast, linezolid significantly suppressed toxin gene expression relative to control at day 2 (adj-p <0.001), then subsequently rose to become statistically indistinguishable from control. As with sigma factors, the pattern of toxin expression was drug-specific. In heatmaps showing unsupervised clustering of toxin gene expression, the samples consistently clustered by drug (Fig. S4A through C), indicating distinct patterns of injury and adaptation. The multifunctional PE and PPE gene category was progressively induced by all drugs (Fig. 3Q). However, heatmaps showing unsupervised clustering demonstrated drug-specific PE and PPE gene expression with samples consistently clustering by drug (Fig. S5A through C). Antibiotics had divergent effects on the expression of DosR genes, a regulon that responds to the inhibition of aerobic respiration (Fig. 3R). Bedaquiline progressively suppressed DosR gene expressions, becoming statistically significantly lower than control at day 14 (adj-p <0.019). Pretomanid did not significantly alter DosR gene expression relative to pre-treatment control at any day. Linezolid progressively induced DosR gene expression, an effect that was significant at days 7 and 14 (adj-p <0.001). Finally, by day 14, bedaquiline and pretomanid significantly induced expression of the insertion sequence and phage category (adj-p <0.001), which is a generalized stress response (Fig. 3S). By contrast, linezolid did not significantly alter the expression of this category relative to control.

### Effect of pairwise drug combinations relative to the effect of monotherapy

Next, we shifted to consider the effect of pairwise drug combinations on the *Mtb* transcriptome after 2, 7, and 14 days of treatment.

#### Combination of bedaquiline and linezolid (BL)

**Fig 4:**
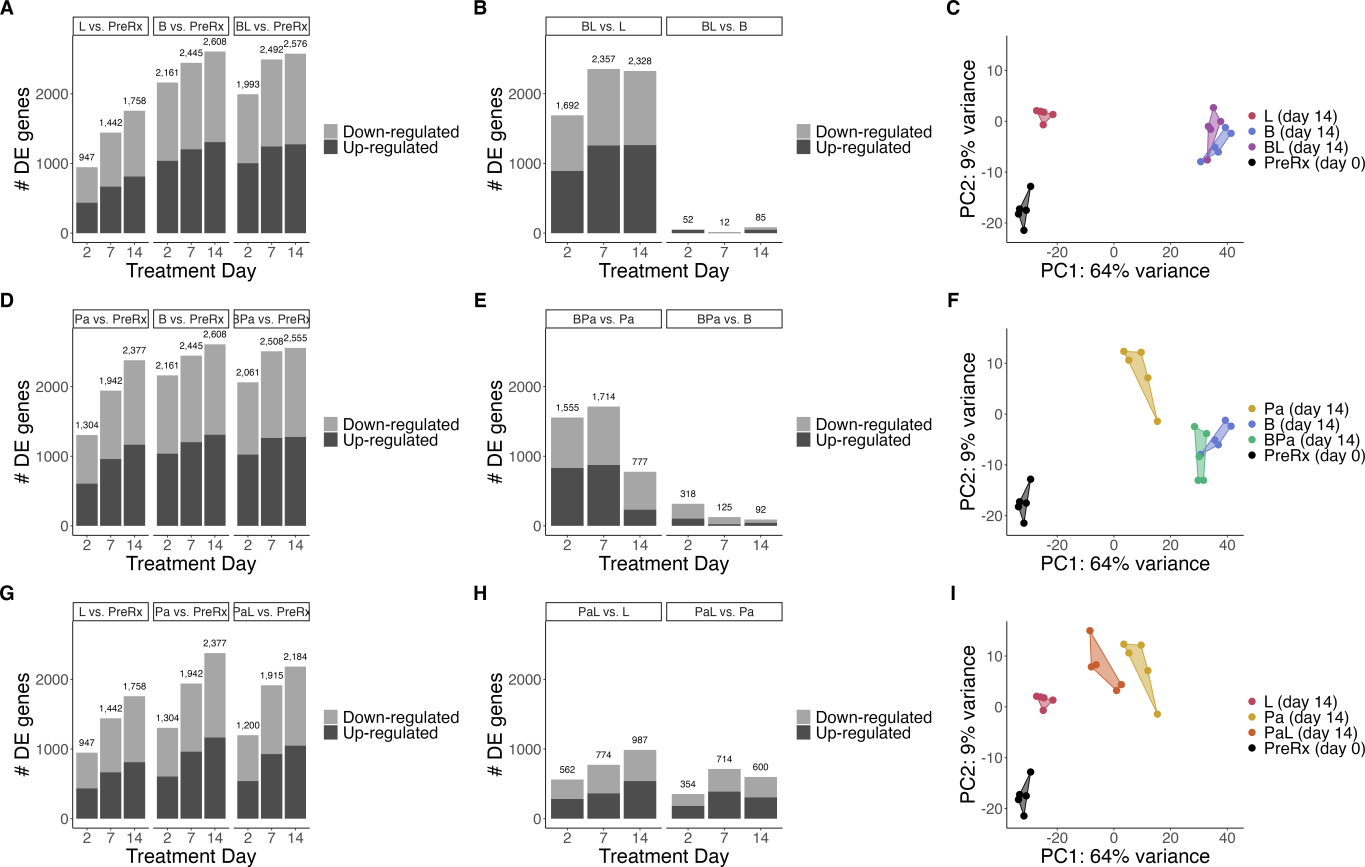
Effect of pairwise drug combinations relative to the effect of monotherapy. (**A–C**) Comparison of gene expression results for bedaquiline (B), linezolid (L), and the combination (BL). (**D–F**) Comparison of gene expression results for bedaquiline (B), pretomanid (Pa), and the combination (BPa). (**G–I**) Comparison of gene expression results for linezolid (L), pretomanid (Pa), and the combination (PaL). (**A**), (**D**), and (**G**) show the number of differentially expressed genes for the two individual antibiotics and their pairwise combination relative to the pretreatment condition. (**B**), (**E**), and (**H**) show the number of differentially expressed genes for the pairwise combination relative to the individual monotherapies. (**C**), (**F**), and (**I**) show the first two principal components of batch-adjusted, VST-normalized gene expression data for the top 500 most variable genes. PCs were calculated across all experimental conditions. Shown are samples from the two individual antibiotics and their pairwise combination at day 14, as well as the pretreatment control measured at day 0. Each point represents an individual sample, and a convex hull highlights each drug condition.

The transcriptional perturbation caused by BL was similar to the effect of bedaquiline alone, as evidenced by the similar number of genes differentially expressed relative to pretreatment control (Fig. 4A), the small number of genes differentially expressed in direct comparison between BL and bedaquiline alone (max = 85 genes differentially expressed at day 14) (Fig. 4B), and the similar transcriptional profiles over time as summarized by the PCA for bedaquiline and BL (Fig. 4C; Fig. S2). By contrast, the effect of BL was highly distinct from the effect of linezolid alone, indicating the dominance of bedaquiline in the combination. This was shown by a substantially larger number of genes differentially expressed relative to pretreatment control for BL than for linezolid (Fig. 4A), a large number of genes differentially expressed in direct comparison between BL and linezolid (Fig. 4B) and distinct transcriptional profiles over time between linezolid and BL, as reflected in the principal components (Fig. 4C; Fig. S2).

#### Combination of bedaquiline and pretomanid (BPa)

The transcriptional change caused by BPa differed modestly from the effect of bedaquiline alone. The number of genes differentially expressed relative to pretreatment control was similar for BPa and bedaquiline alone (Fig. 4D). Following day 2, at which point 316 genes were significant, relatively few genes were differentially expressed in direct comparison between BPa and bedaquiline (Fig. 4E). BPa and bedaquiline had similar transcriptional profiles over time, as seen in the PCA (Fig. 4F; Fig. S2). By contrast, the effect of BPa was highly distinct from the effect of pretomanid alone, again highlighting the dominance of bedaquiline in the combination. This was shown by the substantially larger number of genes differentially expressed relative to pretreatment control for BPa than for pretomanid alone (Fig. 4D), a large number of genes differentially expressed in direct comparison between BPa and pretomanid (Fig. 4E), and the distinct transcriptional profiles over time, as seen in the PCA (Fig. 4F; Fig. S2).

#### Combination of pretomanid and linezolid (PaL)

The transcriptional change induced by PaL was distinct from the effect of either pretomanid or linezolid alone (Fig. 4G through I). For example, on day 14, pretomanid and linezolid had 600 and 987 differentially expressed genes relative to PaL, respectively (Fig. 4H). The transcriptional profile over time of PaL was intermediate between either pretomanid or linezolid, indicating a blended effect.

### Effect of BPaL relative to bedaquiline alone

**Fig 5:**
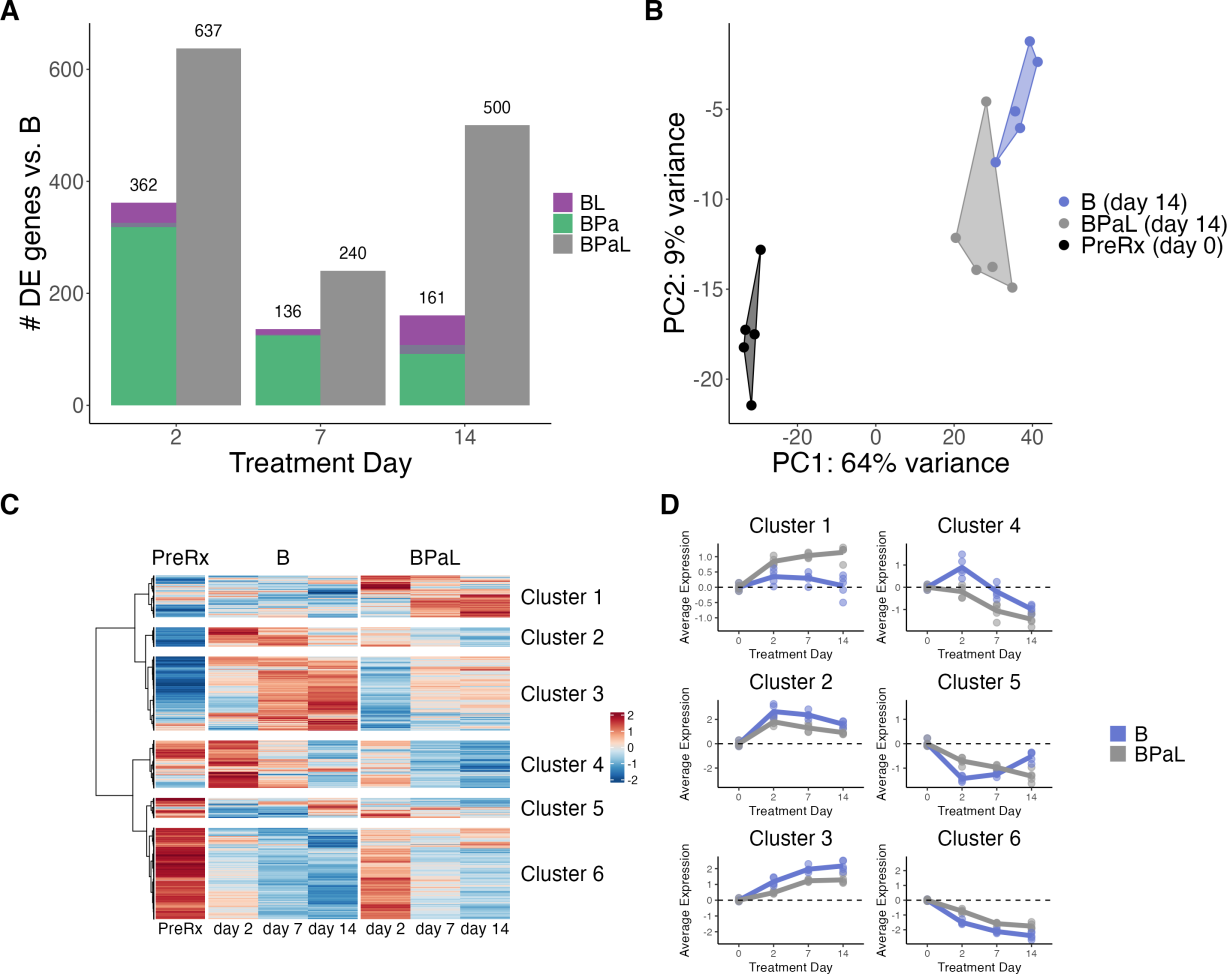
The effect of BPaL relative to bedaquiline alone. (**A**) Number of differentially expressed genes for the pairwise combinations containing bedaquiline (BL and BPa) relative to bedaquiline (**B**) alone, compared with the number of differentially expressed genes of the full BPaL regimen relative to bedaquiline alone. (**B**) The first two principal components of batch-adjusted VST-normalized gene expression data for the top 500 most variable genes. PCs were calculated across all experimental conditions. Shown are samples from B and the BPaL regimen at day 14, as well as the pretreatment control measured at day 0. Each point represents an individual sample, and a convex hull highlights each drug condition. (**C**) Heatmap showing the VST-normalized scaled gene expression averaged across samples for genes differentially expressed between B and BPaL at any time point. Hierarchical clustering identified six broad patterns. (**D**) Average expression for each cluster from plot (**C**) across time points for B and BPaL samples. Each point represents an individual mouse. Lines connect the average value for each treatment across time points. Points and lines are colored by treatment. Values are centered around the average value for the pretreated samples so that points above and below zero represent upregulation and downregulation relative to pretreatment, respectively. Plots for each cluster have unique scales to better show the differences between B and BPaL.

Because preceding analyses indicated that bedaquiline dominates pairwise combinations, we evaluated the effect of adding both pretomanid and linezolid to bedaquiline. We found that PaL modified the effect of bedaquiline synergistically in the sense that the number of genes significantly differentially expressed between BPaL and bedaquiline was greater than expected based on the addition of either drug alone. For example, at day 14, BPa and BL differed from bedaquiline by 92 and 85 genes, respectively, but BPaL differed from bedaquiline by 500 genes (Fig. 5A). Transcriptional synergy was also observed at days 2 and 7. PCA indicates that BPaL led to a distinct longitudinal transcriptional profile relative to bedaquiline alone (Fig. 5B; Fig. S2).

To elucidate the differing effects of BPaL relative to bedaquiline monotherapy, we evaluated the longitudinal change of genes differentially expressed at any time point between BPaL and bedaquiline monotherapy and identified six gene clusters with distinct patterns of change across the two therapies (Fig. 5C and D). Cluster 1 contained genes induced to a greater degree by BPaL than by bedaquiline monotherapy but was not significantly enriched for any gene sets (File S5). Conversely, Clusters 2 and 3 comprised genes that were induced to a lesser degree by BPaL than by bedaquiline monotherapy. Cluster 2 was statistically enriched for genes involved in regulatory functions, including transcription factors (adj-p = 0.003) and repressor activators (adj-p <0.001), suggesting transient reprogramming in response to initial drug stress. Cluster 3 was statistically enriched for phage-related functions (adj-p = 0.003), a non-specific stress response. Cluster 4 contained genes that were initially induced by bedaquiline alone, before falling across later time points, while gradually decreasing in BPaL. Cluster 4 was statistically enriched for regulatory and stress responses, including genes involved in toxin-antitoxin systems (adj-p <0.001) and the enduring hypoxic response (adj-p <0.001). Conversely, Cluster 5 was transiently repressed on day 2 but then rose over time for bedaquiline monotherapy while continuing to decrease for BPaL. Cluster 5 was enriched for three categories related to cholesterol catabolism, including the KstR2 regulon (adj-p <0.001), suggesting that BPaL caused sustained repression, whereas bedaquiline allowed the recovery of this important metabolic function. Cluster 6 was repressed more rapidly and to a greater degree over time by bedaquiline monotherapy than by BPaL. This cluster was enriched for categories related to metabolism (e.g., TCA cycle, adj-p = 0.005), protein synthesis (e.g., ribosomal proteins, adj-p <0.001), and cell wall synthesis (e.g., PDIM, adj-p = 0.023), broadly indicating greater inhibition of activity by bedaquiline alone.

## DISCUSSION

SEARCH-TB is a novel PD marker that evaluates the biological activity of drugs based on *Mtb* physiologic processes rather than *Mtb* burden. Here, we evaluated the capacity of SEARCH-TB to elucidate drug interactions in the BPaL regimen in a mouse TB treatment model. When given in monotherapy, each drug elicited a distinct transcriptional response that evolved over time, consistent with a cascade of progressive physiologic injury and adaptation. When given in pairwise combinations, bedaquiline dominated the effect of either pretomanid or linezolid. When given as a three-drug combination, pretomanid and linezolid appeared to modify the effect of bedaquiline synergistically, producing a transcriptional change greater than would be expected for either drug alone. The SEACH-TB molecular analysis of physiologic processes provided vastly more granular information than CFU alone. This work positions SEARCH-TB as a highly granular pharmacodynamic tool for understanding the effect of drugs and regimens *in vivo*.

These results confirmed and expanded upon our previous study of first-line drugs that identified drug-specific transcriptional changes after one month of treatment in the BALB/c mouse (11) by performing a time-course study and evaluating two drugs not examined previously (pretomanid and linezolid). A first major finding was that the effect of drugs on the *Mtb* transcription increased over time, suggesting a progressive cascade of injury and adaptation that presumably culminates in the loss of homeostasis and cell death. A second major finding was that the effects of bedaquiline, pretomanid, and linezolid were distinct, a phenomenon particularly evident in the evaluation of specific biological processes. For example, bedaquiline suppressed the expression of genes related to metabolism, protein, and cell wall synthesis, active modulation of host response (i.e*.,* ESX1, ESX3, and ESX5 loci), as well as the primary “housekeeping” sigma factor *sigA,* significantly faster and more profoundly than pretomanid or linezolid. Bedaquiline inhibits ATP synthesis. The expression changes we observed indicate that the resulting energy starvation creates a profoundly quiescent, incapacitated phenotype. The pattern of injury and adaptation induced by linezolid was distinct, marked by continuing macromolecule synthesis and less induction of stress responses such as toxins and insertion sequences than bedaquiline or pretomanid. Linezolid induced the expression of certain processes, such as DosR and ESX5, that were suppressed by pretomanid and bedaquiline. Interestingly, pretomanid appeared to “follow” bedaquiline over time such that the day 14 pretomanid transcriptome was similar to the day 2 bedaquiline transcriptome. By contrast, the linezolid transcriptome progressively diverged from both pretomanid and bedaquiline over time. These monotherapy time-course exposure results indicate that each drug’s initial engagement with its molecular target initiates distinct secondary cascades of injury and adaptation. Finally, the monotherapy results highlight that SEARCH-TB reveals drug effects that are indiscernible based on CFUs. In particular, CFUs showed that linezolid had a static effect, suggesting minimal activity. By contrast, SEARCH-TB showed that linezolid altered the expression of thousands of genes, indicating a shift in *Mtb* physiology.

SEARCH-TB for pairwise combinations showed that BL or BPa closely resembled bedaquiline alone. The dominance of the bedaquiline effect is consistent with the central role of bedaquiline in contemporary treatment regimens. By contrast, the combination of pretomanid and linezolid produced a transcriptional response that was intermediate between either drug, demonstrating that each drug modifies the pattern of injury and adaptation induced by the other.

Although the addition of a single drug to bedaquiline had a small effect, the addition of both pretomanid and linezolid to bedaquiline altered the *Mtb* transcriptome more than anticipated based on the results of the addition of either pretomanid or linezolid to bedaquiline individually. Addition of PaL appeared to mitigate the bedaquiline-induced suppression of metabolism, protein synthesis, and cell wall synthesis, indicating less suppression of *Mtb* activity with BPaL. Since a more active phenotype might be more capable of recovery on agar, this may explain the seemingly paradoxical but well-established (31, 32) finding that BPaL decreased CFU counts less than bedaquiline alone. In contrast, other processes exhibited a greater change at the end of treatment with BPaL than with bedaquiline alone. For example, gene sets associated with stress response were more repressed by BPaL than by bedaquiline alone. Additionally, a gene set that decreased more for BPaL than for bedaquiline alone (Cluster 5) was enriched for several cholesterol pathways, suggesting that the addition of PaL further inhibited cholesterol catabolism. Cholesterol catabolism is essential for *Mtb* intracellular survival (36, 37) and is a promising novel drug target (2). Others have previously shown that the cholesterol inhibitor GSK-286 enhanced efficacy to a greater degree when added to BPaL than when added to BPa (38). This highlights a common observation in murine studies: the effect of adding a single drug to a regimen often depends on the specific combination of drugs it is added to (39–41). We hypothesize that the activity of one drug may be conditional on the pattern of injury and adaptation induced by other drugs in the regimen. By elucidating *Mtb* physiology *in vivo,* SEARCH-TB provides a novel basis for understanding these complex drug interactions.

This work adds to the body of evidence (11, 25) that SEARCH-TB is a highly granular and informative PD marker. CFU enumerates the burden of *Mtb* capable of growth on agar but provides no information on bacterial physiologic processes. By contrast, SEARCH-TB provides no information about *Mtb* burden but gives rich information about the cascade of injury and adaptation triggered by drug stress. Here, we found that SEARCH-TB gave a markedly different perspective on drug interactions than CFUs. For example, BPa and BPaL had indistinguishable effects on CFU before day 21. By contrast, SEARCH-TB showed that the addition of linezolid to BPa meaningfully altered *Mtb* physiology.

The process of establishing the practical value of a novel PD marker is a stepwise journey. To date, our SEARCH-TB studies have been descriptive, documenting the effect of HRZE (25), individual drugs (11), and now drug interactions in the BALB/c mouse. Although these initial publications serve as proof of concept, we recognize that the capacity to measure biologic activity in a new way does not in and of itself provide actionable information for drug development. A crucial next step will be linking SEARCH-TB results with relapse outcomes in the BALB/c mouse.

SEARCH-TB could provide practical value for drug evaluation if patterns of injury and adaptation measured by the transcriptome can predict drug activity and/or interactions *in vivo*. The INDIGO-MTB model provided proof of concept that transcriptional responses to drug exposure *in vitro* can predict drug combinations that optimally inhibit growth *in vivo* (42). The next step will be assessing the ability of SEARCH-TB to predict the treatment shortening (i.e*.,* sterilizing activity) of regimens by determining whether specific patterns of injury and adaptation are associated with faster time to non-relapsing cure in mice (i.e*.,* shorter T_95_ in the BALB/c relapsing mouse model). Identifying an early signature of treatment shortening in the BALB/c model could reduce reliance on the long and resource-intensive relapsing mouse model that is a bottleneck in regimen evaluation and initiate a new era of drug and regimen evaluation based on highly granular molecular measures of injury and adaptation rather than crude measures of culturable *Mtb* burden.

This study has several limitations. First, we selected the BALB/c high-dose aerosol infection model because it is the cornerstone of contemporary regimen development. Drug effects in other TB treatment models, such as the C3HeB/FeJ mouse, may differ due to variation in pharmacokinetics and tissue microenvironments. A future direction will be applying SEARCH-TB to evaluate bacterial phenotypes and drug responses in diverse tissue types. Second, as performed here, SEARCH-TB quantified the population-average bacterial transcriptome rather than capturing heterogeneity within the bacterial population. Future studies will evaluate the capacity of the highly sensitive SEARCH-TB method to evaluate gene expression within individual host cells. Finally, although the transcriptome documents the cascade of physiologic change initiated by drug stress, it cannot disentangle what components of this change are functional (i.e*.,* adaptations that enable survival) versus dysfunctional (i.e*.,* “damage” that results from profound physiologic stress).

Using SEARCH-TB allowed us to assess drug interactions within the BPaL regimen *in vivo* based on how they affect physiologic processes. This revealed that individual drugs initiate distinct cascades of injury and adaptation that are modified in complex ways when drugs are combined, resulting in either dominance by a single drug (i.e*.,* bedaquiline) or a blended response. The response to the full three-drug BPaL regimen indicates that higher-order interactions may be more than the sum of parts. Broadly, this work demonstrates the capacity of SEARCH-TB to elucidate drug interactions with vastly greater granularity than the existing conventional measure of culturable *Mtb* burden (CFU), pointing to a future in which optimal combinations could be identified based on *in vivo* molecular measures of *Mtb* physiologic processes.

## MATERIALS AND METHODS

### Murine experiment and sampling

We used RNA samples from a recent publication (32) that fully describes murine procedures. Briefly, using the BALB/c high-dose aerosol infection model, mice were treated via oral gavage with standard doses of individual drugs, pairwise combinations, and the BPaL regimen (Table S1). Mice were humanely euthanized before treatment initiation (pretreatment control, *N* = 5) or following 2, 4, 7, 11, 14, or 21 days of treatment (*N* = 5 per treatment/time point). CFU was quantified, and RNA was preserved and extracted as previously described (32). All animal studies were performed at Colorado State University in a certified animal biosafety level III facility and conducted in accordance with the guidelines of the Colorado State University Institutional Animal Care and Use Committee (reference number: 1212).

### Library preparation and RNA sequencing

We prepared and sequenced RNA from mice euthanized before treatment initiation (pretreatment control) and 2, 7, and 14 days after treatment initiation. Samples were randomized, and libraries were prepared in five different batches. We followed sequencing and quality control methods previously developed for the SEARCH-TB platform (25). Briefly, RNA was reverse-transcribed, cDNA targets were amplified using the custom SEARCH-TB panel, and sequencing was performed using an Illumina NovaSeq6000 device. The sequencing data were processed, and quality control measures were taken using pipelines previously described (25).

### Statistical analysis

To broadly characterize the differences in expression profiles between conditions and time points, we used principal components analysis. First, the data were transformed using the variance stabilizing transformation (VST) from the DESeq2 package (43). To account for the library preparation batch effect, we applied the removeBatchEffect function from the limma package to the normalized data (44). Principal components were then calculated on the full data set using the 500 most variable genes, and these components were used for all PCA plots presented throughout the manuscript to ensure comparability.

We performed differential expression analysis by fitting negative binomial generalized linear models using the edgeR package (45). Models were fit using a term for drug condition/time point, and a term for batch was also included to adjust for batch effect. Likelihood ratio tests were used to assess differences in expression between different conditions at each time point. Genes with Benjamini-Hochberg-adjusted *P*-values (33) of less than 0.05 for tests of interest were deemed significantly differentially expressed.

We performed hierarchical clustering of VST-normalized data, averaged across samples in each condition/time point, to identify groups of genes with similar patterns of expression across time in the monotherapies. For each of the three monotherapies, we first removed invariant genes that were not differentially expressed between any time points and then performed hierarchical clustering using Euclidean distance and Ward’s method (46). A similar analysis was used to identify groups of genes that had differing expression patterns over time across the full BPaL regimen compared with bedaquiline monotherapy. For this analysis, we removed genes that showed no differential expression between bedaquiline and BPaL at any time point.

We performed enrichment analysis to identify functional gene categories that were enriched for genes in clusters identified using hierarchical clustering. We used functional categories established by Cole et al. (34) and curated from the literature (Table S2), excluding categories with three or fewer genes. Enrichment was assessed using the hypergeometric test in the hypeR package (47). Significance was assessed using a Benjamini-Hochberg adjusted *P*-value threshold of 0.05.

We also tested for differences in the average scaled VST-normalized expression of each gene set (using the Cole categories and curated sets described above). For each gene set, we computed the average scaled expression across all genes in the set for each sample, then fit a linear model with condition/time point and batch as covariates. Pairwise comparisons between condition/time point groups were conducted using Tukey’s method to adjust for multiple testing within each gene set. To account for testing across multiple gene sets, we further applied a Benjamini-Hochberg correction across all gene sets for each comparison. Gene sets with adjusted *P*-values < 0.05 were considered significantly differentially expressed.

We visualized the expression patterns for functional gene categories, as well as gene clusters identified using hierarchical clustering, using sample-specific, scaled, batch-corrected VST-normalized expression values averaged across the genes in each gene category/cluster (Fig. S6).

All analyses were performed using R (v4.4.1). Differential expression and visualizations can be explored interactively using an Online Analysis Tool (https://microbialmetrics.org/analysis-tools/).

## Data Availability

All raw sequencing data have been deposited in the Sequence Read Archive (SRA) under BioProject accession PRJNA1240236. Individual samples have BioSample accession numbers SAMN47511482 through SAMN47511591. Code is available via the following link: https://github.com/ewynn610/BPaL_manuscript_code

## SUPPLEMENTAL MATERIAL

Data S1 aac.00492-25-s0001.xlsx BDQ cluster enrichment analysis results.

Data S2 aac.00492-25-s0002.xlsx PA cluster enrichment analysis results.

Data S3 aac.00492-25-s0003.xlsx L cluster enrichment analysis results.

Data S4 aac.00492-25-s0004.xlsx Adjusted *P*-values from tests comparing the differences between average batch adjusted, VST-normalized, and scaled gene expression for key biological gene categories.

Data S5 aac.00492-25-s0005.xlsx BPaL/BDQ cluster enrichment analysis results.

Supplemental material aac.00492-25-s0006.docx Fig S1 to S6; Tables S1 and S2.

ASM does not own the copyrights to Supplemental Material that may be linked to, or accessed through, an article. The authors have granted ASM a non-exclusive, world-wide license to publish the Supplemental Material files. Please contact the corresponding author directly for reuse.
