# Supplementary material for: Deconvoluting drug interactions using M. tuberculosis physiologic processes: transcriptional disaggregation of the BPaL regimen in vivo
Source: Antimicrob Agents Chemother. 2025 Sep 18;69(11):e00492-25. doi: 10.1128/aac.00492-25 (PMC12587609; doi:10.1128/aac.00492-25)
Supplement: Supplemental material — Fig S1 to S6; Tables S1 and S2. [file aac.00492-25-s0006.docx]

**SUPPLEMENTAL INFORMATION**

**Deconvoluting drug interactions based on *Mtb* physiologic processes:**

**Transcriptional disaggregation of the BPaL regimen *in vivo***

Table of Contents

[Supplemental Results 1](#_Toc355564889)

[Figure S1: Change in gene expression over time for Pa and L. 2](#_Toc1228476425)

[Figure S2: Principal components plot for all drugs/combination therapies and timepoints. 2](#_Toc1619930633)

[Figure S3: Heatmaps of expression of sigma factors in monotherapies. 3](#_Toc2146599928)

[Figure S4: Heatmaps of expression of toxins in monotherapies. 3](#_Toc1768197778)

[Figure S5: Heatmaps of expression of PE and PPE families in monotherapies. 4](#_Toc1607584364)

[Supplemental Methods 4](#_Toc340215747)

[Table S1. Antibiotic dosing 5](#_Toc557430866)

[Table S2. Curated gene categories used for enrichment analysis. 5](#_Toc416710061)

[Figure S6: Illustration of the method used to calculate and visualize the average expression of gene sets. 7](#_Toc893950503)

[Supplemental references 8](#_Toc1837257165)

# **Supplemental Results**

## **Figure S1:** Change in gene expression over time for Pa and L.


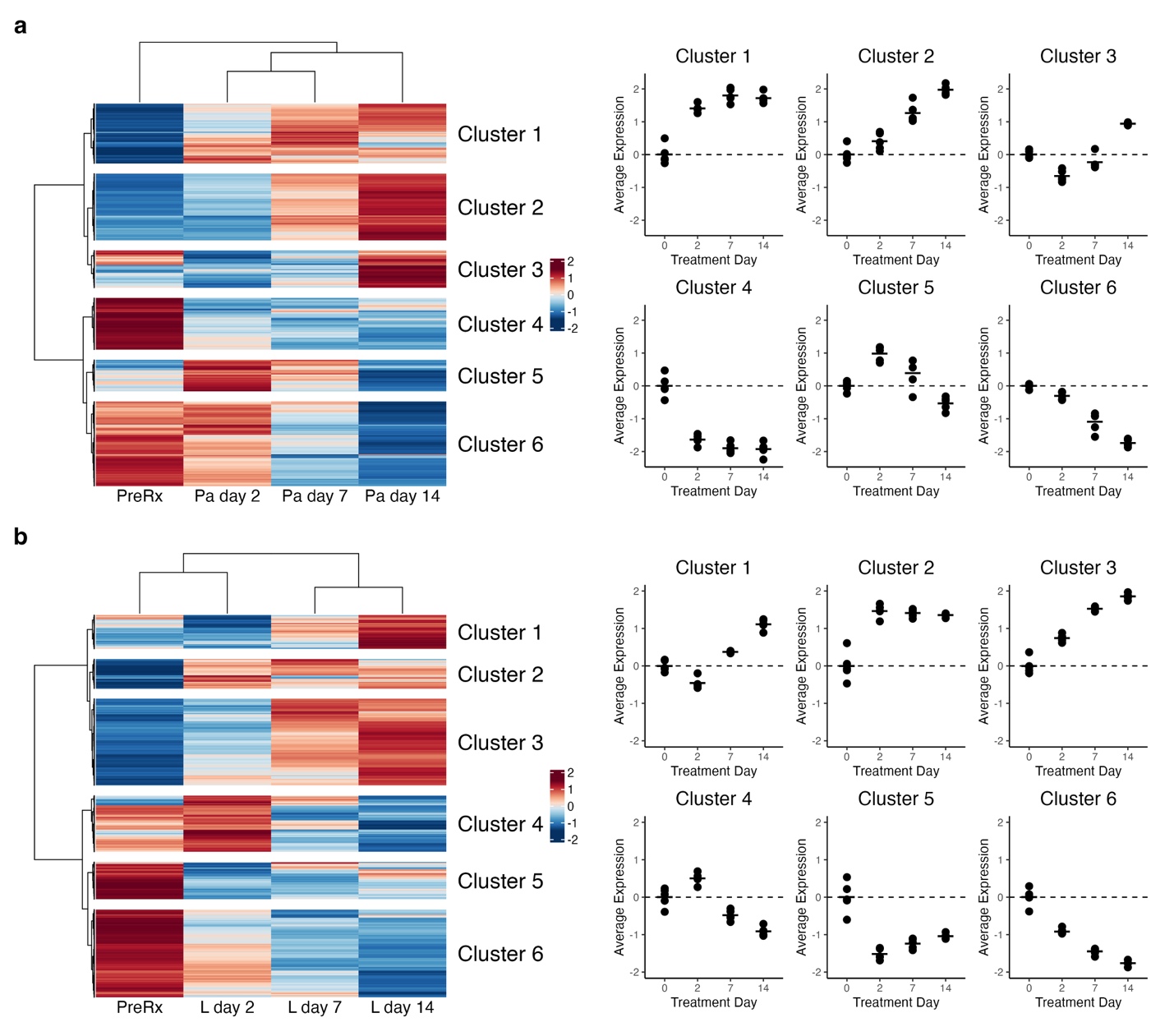


**Figure S1.** Change in gene expression over time for Pa (a) and L (b)**.** Heatmaps show the VST normalized scaled gene expression averaged across samples. Genes that were not differentially expressed between at least two timepoints were excluded. For each monotherapy, hierarchical clustering identified six broad patterns. Dot plots show the average of VST-normalized, scaled expression across timepoints for the clusters. Each point represents an individual mouse. Horizontal lines indicate average values. Values are centered around the average value for the pretreated samples so that points above and below zero represent upregulation and downregulation relative to pretreatment, respectively.

## **Figure S2:** Principal components plot for all drugs/combination therapies and timepoints.


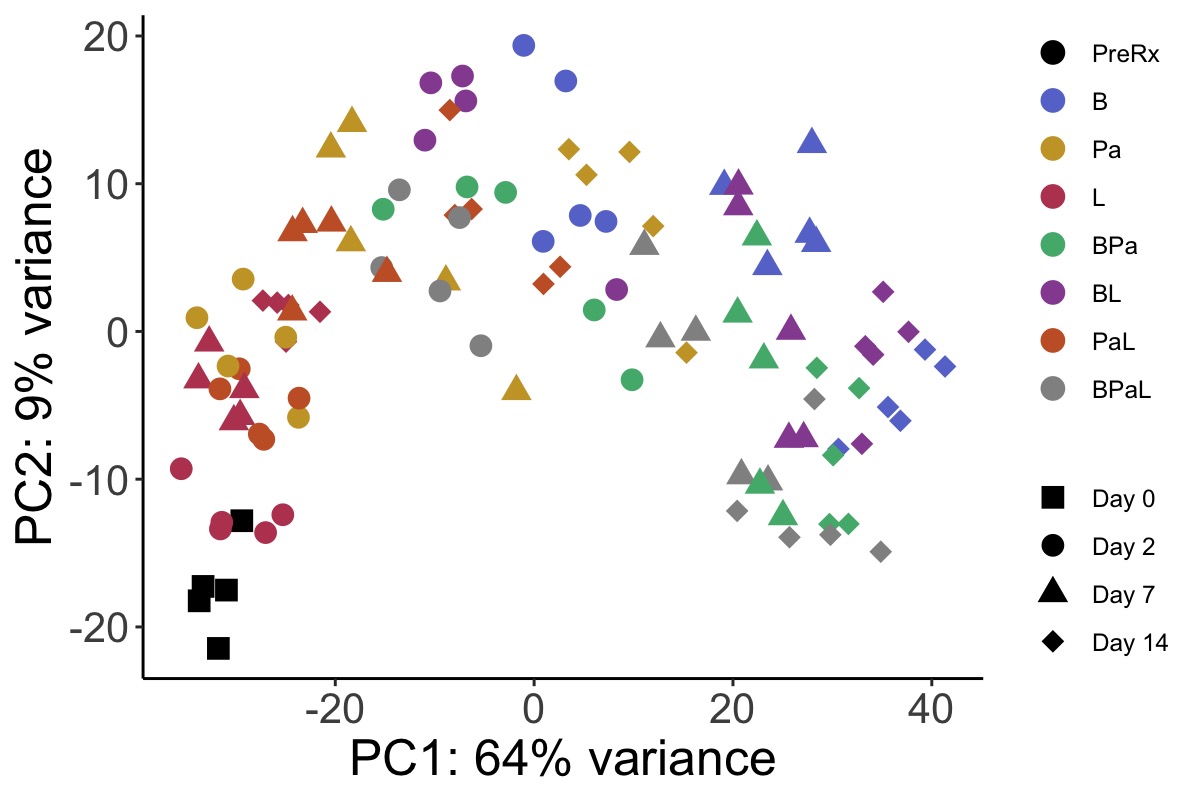
**Figure S2.** The first two principal components of batch adjusted, VST-normalized gene expression data for the top 500 most variable genes. PCs were calculated across all experimental conditions.

## **Figure S3:** Heatmaps of expression of sigma factors in monotherapies.


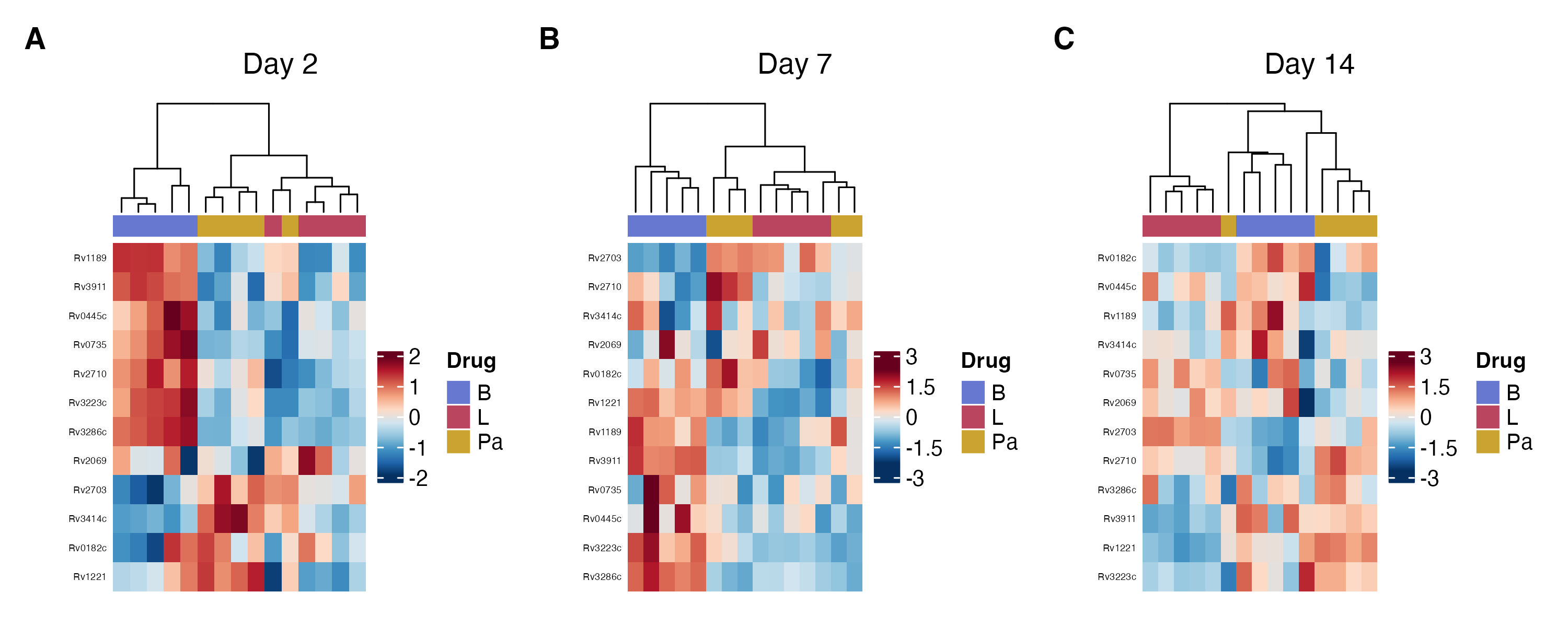
**Figure S3.** Heatmaps of expression of sigma factors in monotherapies. Heatmaps show the batch adjusted, VST-normalized, scaled gene expression of monotherapy samples for sigma factor genes at **(A)** day 2, **(B)** day 7, and **(C)** day 14. Genes and samples are hierarchically clustered for each heatmap and so are not in the same order across timepoints.

## **Figure S4:** Heatmaps of expression of toxins in monotherapies.


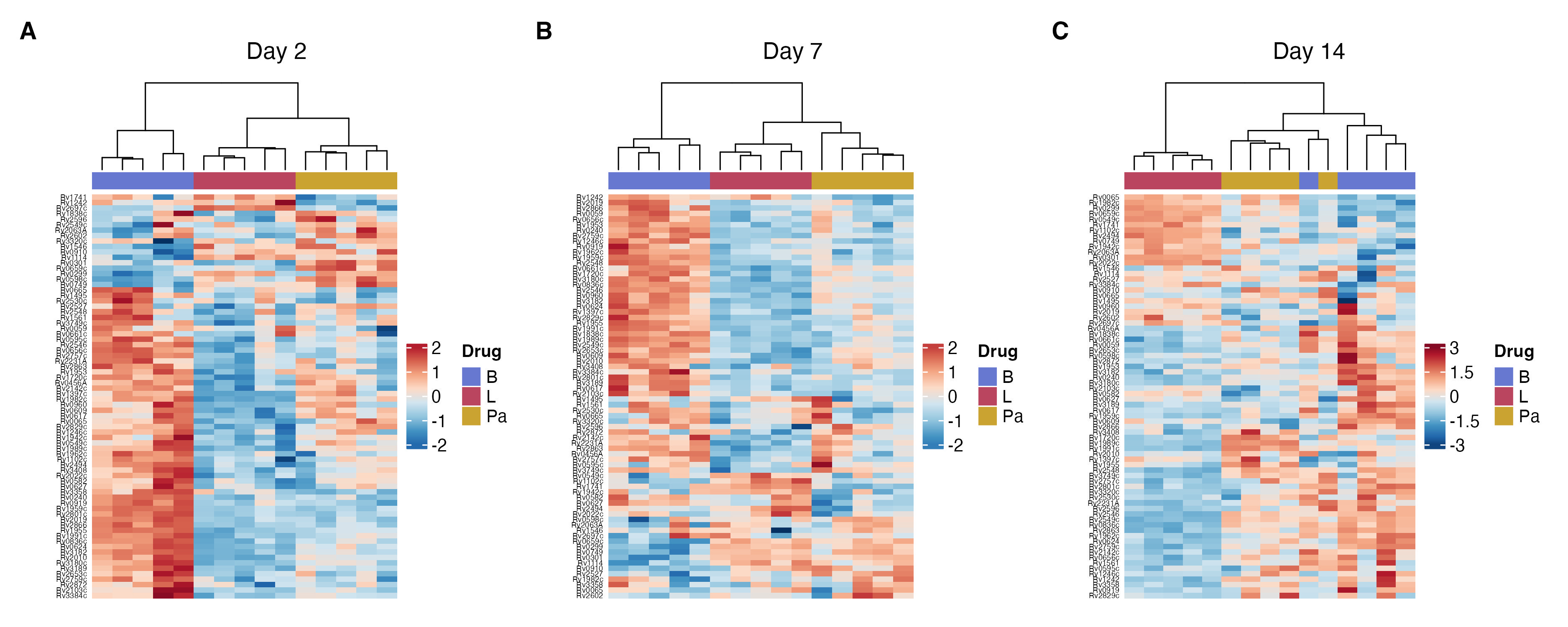


**Figure S4.** Heatmaps of expression of toxins in monotherapies. Heatmaps show the batch adjusted, VST-normalized, scaled gene expression of monotherapy samples for toxin genes at **(A)** day 2, **(B)** day 7, and **(C)** day 14. Genes and samples are hierarchically clustered for each heatmap and so are not in the same order across timepoints.

## **Figure S5:** Heatmaps of expression of PE and PPE families in monotherapies.


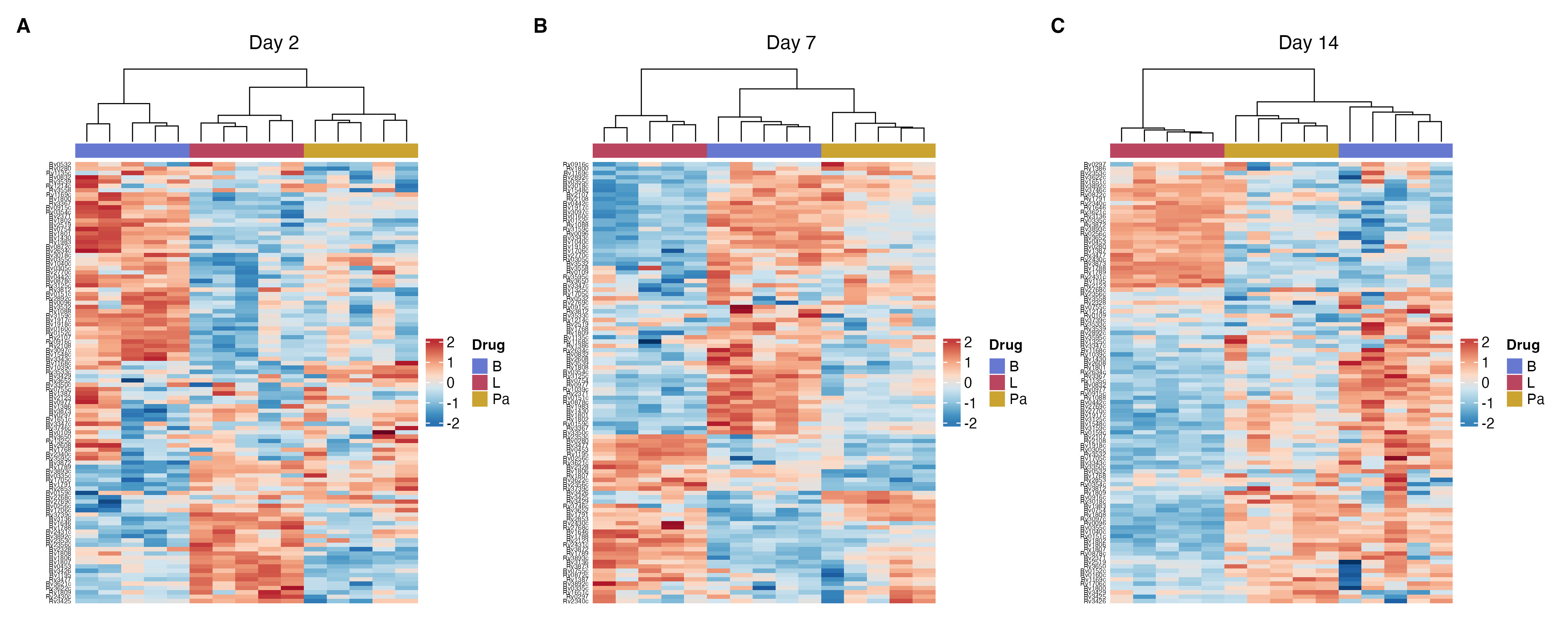


**Figure S5.** Heatmaps of expression of PE and PPE families in monotherapies. Heatmaps show the batch adjusted, VST-normalized, scaled gene expression of monotherapy samples for PE and PPE family genes at **(A)** day 2, **(B)** day 7, and **(C)** day 14. Genes and samples are hierarchically clustered for each heatmap and so are not in the same order across timepoints.

# **Supplemental Methods**

## **Table S1.** Antibiotic dosing

Antibiotic dosage concentration for mice in each antibiotic treatment group. Mice began treatment 11 days post infection via oral gavage seven days a week.

| **Treatment** | **Dose (mg/kg)** |
| --- | --- |
| Bedaquiline | 25 |
| Linezolid | 100 |
| Pretomanid | 50 |

## **Table S2.** Curated gene categories used for enrichment analysis.

Manuscript figures and statistical functional enrichment analysis used sets of biologically linked genes curated from the literature. The number of genes in category indicates the number of genes identified in the literature source. Since SEARCH-TB quantifies expression of 89% of *Mtb* transcripts, not all genes in each category were quantified. Number of genes in SEARCH-TB indicates the number assayed.

| **Category** | **# of Genes in SEARCH-TB** | **# of Genes in Category** | **Source** |
| --- | --- | --- | --- |
| ABC transporters - Type I peptide and amino acids | 6 | 8 | (Soni, Dubey, & Bhatnagar, 2020)^1^ |
| ABC transporters - Type I Sugar Import | 12 | 12 | (Soni, Dubey, & Bhatnagar, 2020)^1^ |
| ABC transporters - Type I anion | 7 | 7 | (Soni, Dubey, & Bhatnagar, 2020)^1^ |
| ABC transporters - Type I phosphate | 8 | 8 | (Soni, Dubey, & Bhatnagar, 2020)^1^ |
| ABC transporters - Type II metal | 5 | 5 | (Soni, Dubey, & Bhatnagar, 2020)^1^ |
| Alternative ribosomal proteins | 4 | 5 | (Prisic et al., 2015)^2^ |
| Antigen 85 | 3 | 3 | (Karbalaei Zadeh Babaki, Soleimanpour, & Rezaee, 2017)^3^ |
| Antitoxins | 72 | 76 | (Shao et al., 2011)^4^ |
| Arabinogalactan (AG) | 18 | 19 | (Abrahams & Besra, 2018)^5^ |
| Beta Oxidation | 18 | 18 | (Schnappinger et al., 2003)^6^ |
| Cell wall synthesis | 40 | 40 | (Kirksey et al., 2011)^7^ |
| Cholesterol A and B ring degradation | 10 | 10 | (Pawełczyk et al., 2021)^8^ |
| Cholesterol C and D ring degradation | 5 | 5 | (Pawełczyk et al., 2021)^8^ |
| Cholesterol side chain degradation | 33 | 33 | (Pawełczyk et al., 2021)^8^ |
| Cutinase-Like Proteins (CULP) | 7 | 7 | (Tallman, Levine, & Beatty, 2016)^9^ |
| Cytochrome oxidase bccaa3 | 6 | 7 | (Lee, Sviriaeva, & Pethe, 2020)^10^ |
| Cytochrome oxidase bd | 3 | 4 | (Lee, Sviriaeva, & Pethe, 2020)^10^ |
| DNA replication and repair | 25 | 27 | (Ditse, Lamers, & Warner, 2017)^11^ |
| DosR | 48 | 48 | (Voskuil et al., 2003)^12^ |
| Drug targets | 40 | 40 | (“Working Group for New TB Drugs.,” 2021)^13^  (Shetye, Franzblau, & Cho, 2020)^14^ |
| Efflux Pumps and Transports | 25 | 26 | (Remm, Earp, Dick, Dartois, & Seeger, 2022)^15^ |
| Enduring Hypoxic Response | 149 | 161 | (Rustad, Harrell, Liao, & Sherman, 2008)^16^ |
| Esterases (Lip family) | 20 | 22 | (Tallman, Levine, & Beatty, 2016)^9^ |
| Esterases (non-Lip family) | 13 | 13 | (Tallman, Levine, & Beatty, 2016)^9^ |
| ESX1 | 18 | 19 | (Gröschel, Sayes, Simeone, Majlessi, & Brosch, 2016)^17^ |
| ESX2 | 12 | 12 | (Gröschel, Sayes, Simeone, Majlessi, & Brosch, 2016)^17^ |
| ESX3 | 9 | 11 | (Gröschel, Sayes, Simeone, Majlessi, & Brosch, 2016)^17^ |
| ESX4 | 7 | 7 | (Gröschel, Sayes, Simeone, Majlessi, & Brosch, 2016)^17^ |
| ESX5 | 11 | 15 | (Gröschel, Sayes, Simeone, Majlessi, & Brosch, 2016)^17^ |
| Fatty Acid Synthases I | 1 | 1 | (Cole et al., 1998)^18^ |
| Fatty Acid Synthases II | 8 | 9 | (Duan, Xiang, & Xie, 2014)^19^ |
| Fumarate reductase | 4 | 4 | (Cole et al., 1998)^18^ |
| Kas operon | 3 | 5 | (Slayden & Barry, 2002)^20^ |
| kstR1 regulon | 70 | 71 | (Wipperman, Sampson, & Thomas, 2014)^21^ |
| kstR2 regulon | 14 | 15 | (Wipperman, Sampson, & Thomas, 2014)^21^ |
| LpqY-SugA-SugB-Sug trehalose transporter | 5 | 5 | (Soni, Dubey, & Bhatnagar, 2020)^1^ |
| Mce1 | 7 | 7 | (Cole et al., 1998)^18^ |
| Mce2 | 6 | 7 | (Cole et al., 1998)^18^ |
| Mce3 | 7 | 7 | (Cole et al., 1998)^18^ |
| Mce4 | 7 | 7 | (Cole et al., 1998)^18^ |
| LAM | 14 | 15 | (Batt, Burke, Moorey, & Besra, 2020)^22^ |
| mmpL | 14 | 14 | (Domenech, Reed, & Barry, 2005)^23^ |
| mmpS | 5 | 5 | (Melly & Purdy, 2019)^24^ |
| Mycobactin Biogenesis | 10 | 10 | (Quadri, Sello, Keating, Weinreb, & Walsh, 1998)^25^ |
| Mycolic acid condensation and transfer | 7 | 7 | (Marrakchi, Lanéelle, & Daffé, 2014)^26^ |
| Mycolic acid modification | 12 | 12 | (Marrakchi, Lanéelle, & Daffé, 2014)^26^ |
| NADH dehydrogenase type I | 12 | 14 | (Cook, Hards, Vilchèze, Hartman, & Berney, 2014)^27^ |
| NADH dehydrogenase type II | 2 | 2 | (Cook, Hards, Vilchèze, Hartman, & Berney, 2014)^27^ |
| Nitrate import and reductase | 6 | 8 | (Cole et al., 1998)^18^ |
| Oxidative Stress | 48 | 49 | (Voskuil, Bartek, Visconti, & Schoolnik, 2011)^28^ |
| PDIM | 20 | 20 | (Rens, Chao, Sexton, Tocheva, & Av-Gay, 2021)^29^ |
| Peptidoglycan (PG) | 32 | 34 | (Maitra et al., 2019)^30^ |
| Phospholipase C | 4 | 4 | (Raynaud et al., 2002)^31^ |
| Primary ribosomal proteins | 50 | 53 | (Prisic et al., 2015)^2^, (Cole et al., 1998)^18^* |
| Sigma Factors | 12 | 13 | (Lew, Kapopoulou, Jones, & Cole, 2011)^32^ |
| Stringent Response - Induced | 58 | 70 | (Dahl et al., 2003)^33^ |
| Stringent Response - Repressed | 66 | 78 | (Dahl et al., 2003)^33^ |
| Succinate dehydrogenase I and II | 7 | 7 | (Hartman et al., 2014)^34^ |
| Toxin-Antitoxin | 146 | 152 | (Shao et al., 2011)^4^ |
| Toxins | 74 | 76 | (Shao et al., 2011)^4^ |
| Transcription Factors | 187 | 198 | (Lew, Kapopoulou, Jones, & Cole, 2011)^32^ |
| Trehalose | 10 | 10 | (Wilson et al., 1999)^35^ |
| Triacylglycerol Synthases | 14 | 16 | (Thanna & Sucheck, 2016)^36^ |
| UgpABCE glycerophosphocholine transporter | 4 | 4 | (Soni, Dubey, & Bhatnagar, 2020)^1^ |
| Universal stress proteins | 9 | 10 | (Lew, Kapopoulou, Jones, & Cole, 2011)^32^ |
| UspABC amino sugar transporter | 3 | 3 | (Soni, Dubey, & Bhatnagar, 2020)^1^ |
| WhiB-Like transcription factors | 7 | 7 | (Wan et al., 2021)^37^ |
| Zur regulon | 17 | 20 | (Dow et al., 2021)^38^ |

* Primary ribosomal proteins category was created by removing the four alternative proteins identified in Prisic et al., 2015 from the “Ribosomal protein and synthesis” gene list identified in Cole et al., 1998.

## **Figure S6:** Illustration of the method used to calculate and visualize the average expression of gene sets.


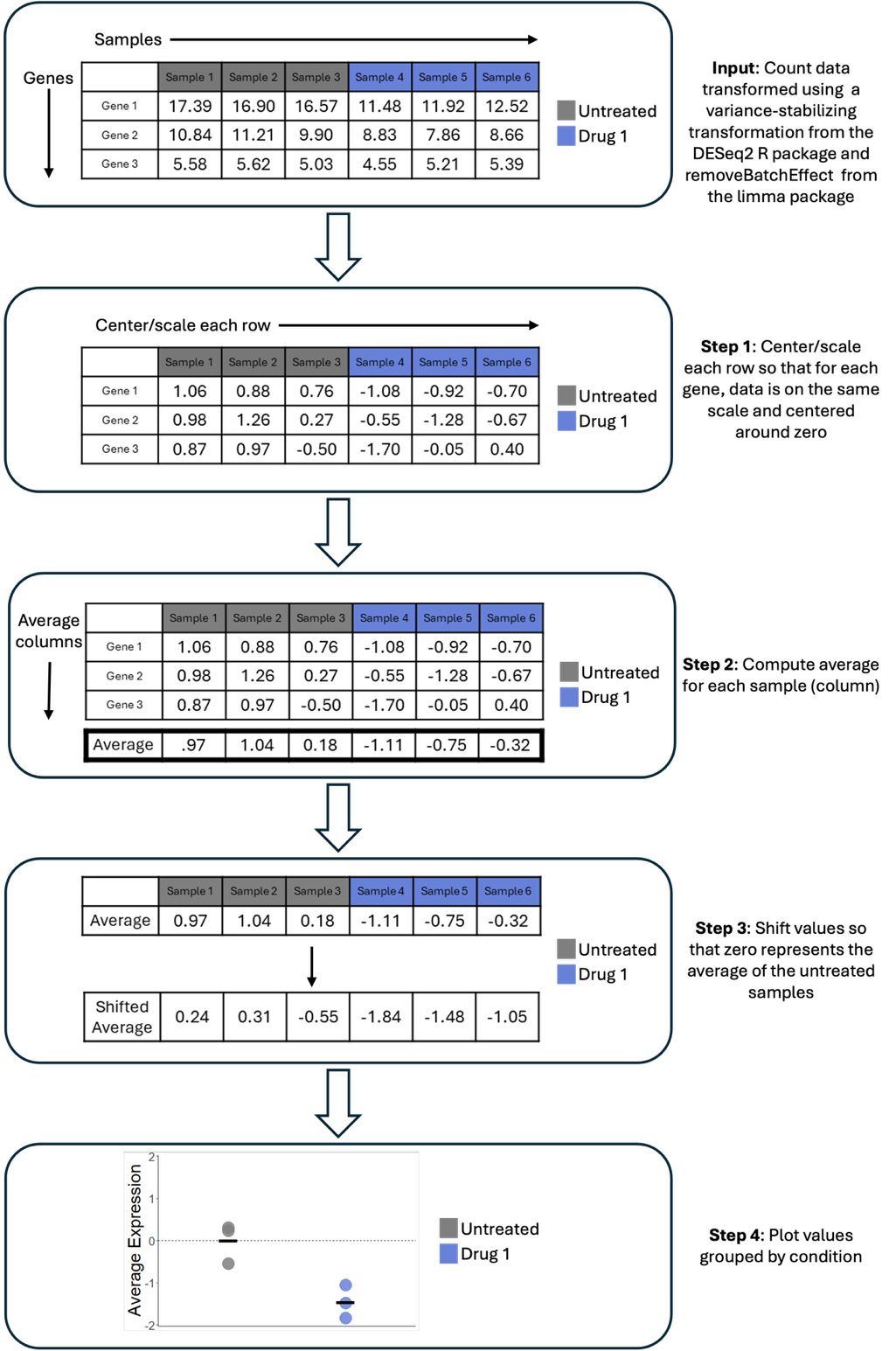


**Figure S6.** Illustration of the method for calculating and plotting the average expression of sets of genes. Mock data from two conditions (untreated and drug 1), each with three samples, is used for demonstration. For simplicity, a gene set of three genes is used. The variance-stabilized data for each gene (batch adjusted using removeBatchEffect function from the limma package in R) is first scaled and centered around zero to ensure comparability. Then, the expression values are averaged for each sample and adjusted so that zero represents the average of the untreated samples. Finally, these values are plotted, providing a comparison of gene expression between conditions for the gene sets of interest and illustrating the spread of gene expression across samples.

# **Supplemental references**

1. Soni, D. K., Dubey, S. K. & Bhatnagar, R. ATP-binding cassette (ABC) import systems of *Mycobacterium tuberculosis*: target for drug and vaccine development. *Emerg Microbes Infect* **9**, 207–220 (2020).

2. Prisic, S. *et al.* Zinc regulates a switch between primary and alternative S18 ribosomal proteins in *Mycobacterium tuberculosis*. *Mol Microbiol* **97**, 263–280 (2015).

3. Karbalaei Zadeh Babaki, M., Soleimanpour, S. & Rezaee, S. A. Antigen 85 complex as a powerful *Mycobacterium tuberculosis* immunogene: Biology, immune-pathogenicity, applications in diagnosis, and vaccine design. *Microb Pathog* **112**, 20–29 (2017).

4. Shao, Y. *et al.* TADB: A web-based resource for Type 2 toxin-antitoxin loci in bacteria and archaea. *Nucleic Acids Res* **39**, D606–D611 (2011).

5. Abrahams, K. A. & Besra, G. S. Mycobacterial cell wall biosynthesis: A multifaceted antibiotic target. *Parasitology* vol. 145 116–133 Preprint at https://doi.org/10.1017/S0031182016002377 (2018).

6. Schnappinger, D. *et al.* Transcriptional adaptation of *Mycobacterium tuberculosis* within macrophages: Insights into the phagosomal environment. *Journal of Experimental Medicine* **198**, 693–704 (2003).

7. Kirksey, M. A. *et al.* Spontaneous phthiocerol dimycocerosate-deficient variants of *Mycobacterium tuberculosis* are susceptible to gamma interferon-mediated immunity. *Infect Immun* **79**, 2829–2838 (2011).

8. Pawełczyk, J. *et al.* Cholesterol-dependent transcriptome remodeling reveals new insight into the contribution of cholesterol to *Mycobacterium tuberculosis* pathogenesis. *Sci Rep* **11**, 1–16 (2021).

9. Tallman, K. R., Levine, S. R. & Beatty, K. E. Small-molecule probes reveal esterases with Persistent Activity in dormant and reactivating *Mycobacterium tuberculosis*. *ACS Infect Dis* **2**, 936–944 (2016).

10. Lee, B. S., Sviriaeva, E. & Pethe, K. Targeting the cytochrome oxidases for drug development in mycobacteria. *Prog Biophys Mol Biol* **152**, 45–54 (2020).

11. Ditse, Z., Lamers, M. H. & Warner, D. F. DNA replication in *Mycobacterium tuberculosis*. *Microbiol Spectr* **5**, (2017).

12. Voskuil, M. I. *et al.* Inhibition of respiration by nitric oxide induces a *Mycobacterium tuberculosis* dormancy program. *Journal of Experimental Medicine* **198**, 705–713 (2003).

13. Working Group for New TB Drugs. https://www.newtbdrugs.org/pipeline/drug-targets (2021).

14. Shetye, G. S., Franzblau, S. G. & Cho, S. New tuberculosis drug targets, their inhibitors, and potential therapeutic impact. *Translational Research* **220**, 68–97 (2020).

15. Remm, S., Earp, J. C., Dick, T., Dartois, V. & Seeger, M. A. Critical discussion on drug efflux in *Mycobacterium tuberculosis*. *FEMS Microbiol Rev* **46**, 1–15 (2022).

16. Rustad, T. R., Harrell, M. I., Liao, R. & Sherman, D. R. The enduring hypoxic response of *Mycobacterium tuberculosis*. *PLoS One* **3**, e1502 (2008).

17. Gröschel, M. I., Sayes, F., Simeone, R., Majlessi, L. & Brosch, R. ESX secretion systems: Mycobacterial evolution to counter host immunity. *Nat Rev Microbiol* **14**, 677–691 (2016).

18. Cole, S. T. *et al.* Erratum: Deciphering the biology of *Mycobacterium tuberculosis* from the complete genome sequence. *Nature* **396**, 190 (1998).

19. Duan, X., Xiang, X. & Xie, J. Crucial components of mycobacterium type II fatty acid biosynthesis (Fas-II) and their inhibitors. *FEMS Microbiol Lett* **360**, 87–99 (2014).

20. Slayden, R. A. & Barry, C. E. The role of KasA and KasB in the biosynthesis of meromycolic acids and isoniazid resistance in Mycobacterium tuberculosis. *Tuberculosis* **82**, 149–160 (2002).

21. Wipperman, M. F., Sampson, N. S. & Thomas, S. T. Pathogen roid rage: Cholesterol utilization by *Mycobacterium tuberculosis*. *Critical Reviews in Biochemistry and Molecular Biology* vol. 49 269–293 Preprint at https://doi.org/10.3109/10409238.2014.895700 (2014).

22. Batt, S. M., Burke, C. E., Moorey, A. R. & Besra, G. S. Antibiotics and resistance: The two-sided coin of the mycobacterial cell wall. *Cell Surface* **6**, 100044 (2020).

23. Domenech, P., Reed, M. B. & Barry, C. E. Contribution of the *Mycobacterium tuberculosis* MmpL protein family to virulence and drug resistance. *Infect Immun* **73**, 3492–3501 (2005).

24. Melly, G. & Purdy, G. E. Mmpl proteins in physiology and pathogenesis of m. Tuberculosis. *Microorganisms* **7**, (2019).

25. Quadri, L. E., Sello, J., Keating, T. A., Weinreb, P. H. & Walsh, C. T. Identification of a *Mycobacterium tuberculosis* gene cluster encoding the biosynthetic enzymes for assembly of the virulence-conferring siderophore mycobactin. *Chem Biol* **5**, 631–645 (1998).

26. Marrakchi, H., Lanéelle, M. A. & Daffé, M. Mycolic acids: Structures, biosynthesis, and beyond. *Chemistry and Biology* vol. 21 67–85 Preprint at https://doi.org/10.1016/j.chembiol.2013.11.011 (2014).

27. Cook, G. M., Hards, K., Vilchèze, C., Hartman, T. & Berney, M. Energetics of respiration and oxidative phosphorylation in mycobacteria. *Microbiol Spectr* **2**, (2014).

28. Voskuil, M. I., Bartek, I. L., Visconti, K. & Schoolnik, G. K. The response of *Mycobacterium tuberculosis* to reactive oxygen and nitrogen species. *Front Microbiol* **2**, 105 (2011).

29. Rens, C., Chao, J. D., Sexton, D. L., Tocheva, E. I. & Av-Gay, Y. Roles for phthiocerol dimycocerosate lipids in *Mycobacterium tuberculosis* pathogenesis. *Microbiology (United Kingdom)* **167**, 001042 (2021).

30. Maitra, A. *et al.* Cell wall peptidoglycan in *Mycobacterium tuberculosis*: An Achilles’ heel for the TB-causing pathogen. *FEMS Microbiol Rev* **43**, 548–575 (2019).

31. Raynaud, C. *et al.* Phospholipases C are involved in the virulence of Mycobacterium tuberculosis. *Mol Microbiol* **45**, 203–217 (2002).

32. Lew, J. M., Kapopoulou, A., Jones, L. M. & Cole, S. T. TubercuList - 10 years after. *Tuberculosis* **91**, 1–7 (2011).

33. Dahl, J. L. *et al.* The role of RelMtb-mediated adaptation to stationary phase in long-term persistence of *Mycobacterium tuberculosis* in mice. *Proc Natl Acad Sci U S A* **100**, 10026–10031 (2003).

34. Hartman, T. *et al.* Succinate Dehydrogenase is the Regulator of Respiration in Mycobacterium tuberculosis. *PLoS Pathog* **10**, (2014).

35. Wilson, M. *et al.* Exploring drug-induced alterations in gene expression in *Mycobacterium tuberculosis* by microarray hybridization. *Proc Natl Acad Sci U S A* **96**, 12833–12838 (1999).

36. Thanna, S. & Sucheck, S. J. Targeting the trehalose utilization pathways of *Mycobacterium tuberculosis*. *Medchemcomm* **7**, 69–85 (2016).

37. Wan, T. *et al.* Structural insights into the functional divergence of WhiB-like proteins in Mycobacterium tuberculosis. *Mol Cell* **81**, 2887-2900.e5 (2021).

38. Dow, A. *et al.* Zinc limitation triggers anticipatory adaptations in *Mycobacterium tuberculosis*. *PLoS Pathog* **17**, e1009570 (2021).
